# Treatment of Rare Inflammatory Kidney Diseases: Drugs Targeting the Terminal Complement Pathway

**DOI:** 10.3389/fimmu.2020.599417

**Published:** 2020-12-10

**Authors:** Marion Anliker-Ort, Jasper Dingemanse, John van den Anker, Priska Kaufmann

**Affiliations:** ^1^ Department of Clinical Pharmacology, Idorsia Pharmaceuticals Ltd, Allschwil, Switzerland; ^2^ Pediatric Pharmacology and Pharmacometrics, University Children’s Hospital Basel (UKBB), University of Basel, Basel, Switzerland; ^3^ Division of Clinical Pharmacology, Children’s National Hospital, Washington, DC, United States

**Keywords:** complement system, C5 antagonist, C5aR1 antagonist, IgA nephropathy, aHUS, ANCA-associated vasculitis, lupus nephritis, C3 glomerulopathy

## Abstract

The complement system comprises the frontline of the innate immune system. Triggered by pathogenic surface patterns in different pathways, the cascade concludes with the formation of a membrane attack complex (MAC; complement components C5b to C9) and C5a, a potent anaphylatoxin that elicits various inflammatory signals through binding to C5a receptor 1 (C5aR1). Despite its important role in pathogen elimination, priming and recruitment of myeloid cells from the immune system, as well as crosstalk with other physiological systems, inadvertent activation of the complement system can result in self-attack and overreaction in autoinflammatory diseases. Consequently, it constitutes an interesting target for specialized therapies. The paradigm of safe and efficacious terminal complement pathway inhibition has been demonstrated by the approval of eculizumab in paroxysmal nocturnal hematuria. In addition, complement contribution in rare kidney diseases, such as lupus nephritis, IgA nephropathy, atypical hemolytic uremic syndrome, C3 glomerulopathy, or antineutrophil cytoplasmic antibody-associated vasculitis has been demonstrated. This review summarizes the involvement of the terminal effector agents of the complement system in these diseases and provides an overview of inhibitors for complement components C5, C5a, C5aR1, and MAC that are currently in clinical development. Furthermore, a link between increased complement activity and lung damage in severe COVID-19 patients is discussed and the potential for use of complement inhibitors in COVID-19 is presented.

## Introduction

The complement system, as part of the innate immune system (1), comprises several important functions beyond fighting microbial infections, which was assumed to be the only role in the early days following its discovery (2). Nowadays, the complement system is known for being a critical part in various pathways of the immune system, such as opsonization of pathogens or damaged host cells, chemotaxis, modulating smooth muscle contraction and vascular permeability (3), pathogen lysis, removal of immune complexes, provision of proliferative signals for adaptive immune cells (4), angiogenesis, tissue regeneration, wound healing, fibrosis, or lipid metabolism (5).

**Figure 1:**
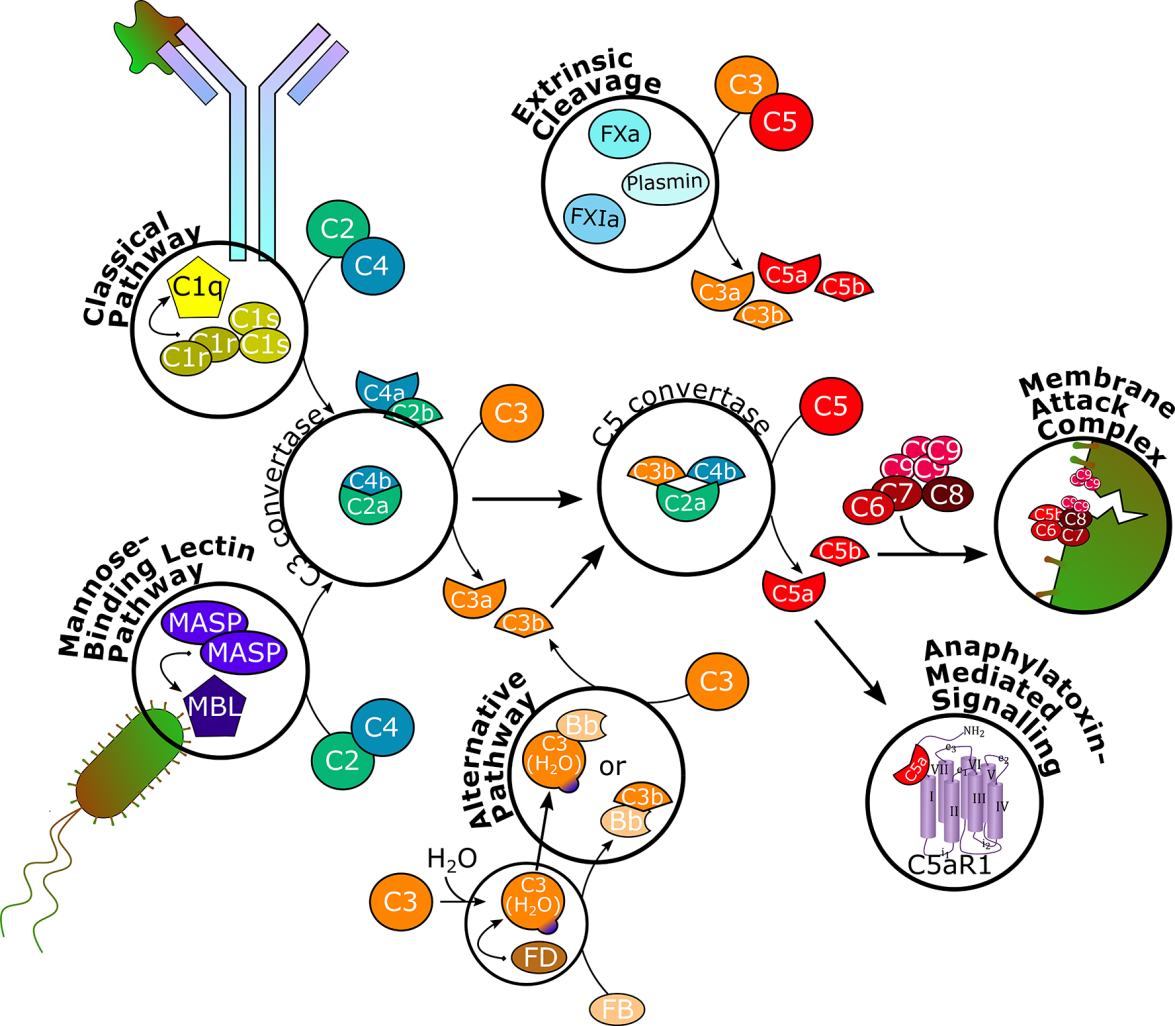
Schematic overview of the complement cascade. The classical pathway is initiated upon binding to antibody-antigen complexes and the mannose-binding lectin pathway is triggered by mannose residues on foreign surfaces. Through recruitment of serine proteases and splitting of C2 and C4, C3 convertase is formed, which splits C3 into C3a and C3b. The alternative pathway is constantly hydrolyzing and cleaving C3 on a low level to allow for rapid self-amplification of the signal. C3b associates with C3 convertase to form C5 convertase, which splits C5 into C5a and C5b. Serine proteases of the coagulation system can independently cleave C3 and C5. C5b recruits C6, C7, C8, and several C9 units to form the membrane attack complex, which inserts into cell membranes to induce cell lysis. C3a and C5a bind to their respective receptors C3aR and C5aR1 to execute various effector functions in different physiological systems. C5aR1, C5a receptor 1; MASP, MBL-associated serine protease; MBL, mannose-binding lectin; FB, factor B; FD, factor D; FXa, factor Xa; FXIa, factor XIa.

Overall, the complement system (**Figure 1**) is a key player in host defense with a strong influence on many other physiological systems, such as the coagulation cascade (6). However, the potential for inadequate regulation or aberrant activity also exists, which can tip the equilibrium towards self-attack and, consequently, contribute to disease (7). Especially the anaphylatoxin C5a has been associated with inflammatory disorders (8, 9), in which the kidney is particularly vulnerable (10). For many years, treatment of rare inflammatory kidney diseases consisted of supportive measures, such as plasma exchange (11), or strong nonspecific immunosuppression, including corticosteroids, which is limited by underlying toxicity and adverse events (12), and many patients progressed to end-stage renal disease (ESRD) requiring dialysis a few years after diagnosis (13, 14).

**Figure 2:**
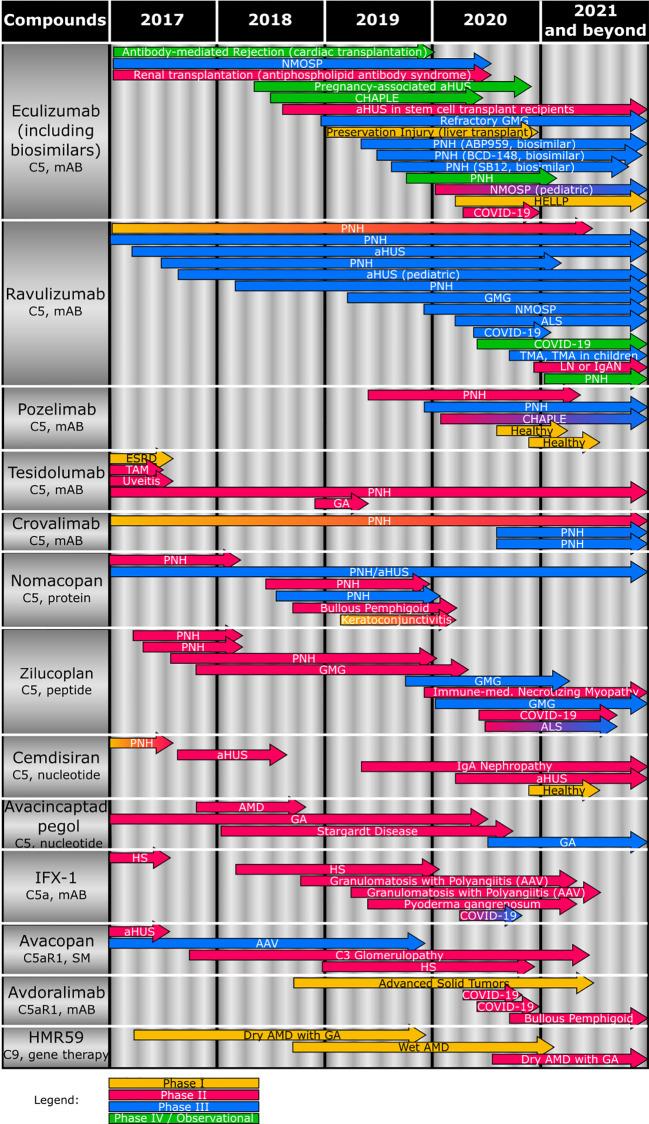
Overview of the clinical status of compounds (including target and compound class) targeting terminal complement effector functions. Studies completed or terminated before 2017 are not depicted. AAV, antineutrophil cytoplasmic antibody-associated vasculitis; aHUS, atypical hemolytic uremic syndrome; ALS, amyotrophic lateral sclerosis; AMD, age-related macular degeneration; CHAPLE, complement hyperactivation, angiopathic thrombosis, protein losing enteropathy; COVID-19, coronavirus disease 2019; ESRD, end-stage renal disease; GA, geographic atrophy; GMG, generalized myasthenia gravis; HELLP, hemolysis, elevated liver enzyme, low platelet; HS, hidradenitis suppurativa; IgAN, IgA nephropathy; IPCV, idiopathic polypoidal choroidal vasculopathy; mAB, monoclonal antibody; NMOSP, neuromyelitis optica spectrum disorder; PNH, paroxysmal nocturnal hemoglobinuria; SM, small molecule; TAM, Transplant-associated microangiopathy.

Of the plethora of molecules involved in the complement cascade, a growing number has been found to be druggable in recent years. This review focuses on the terminal effector function, i.e., C5 and corresponding cleavage products, and aims at providing an overview on rare inflammatory kidney diseases with complement contribution and the current status of compounds in clinical development that target the terminal pathway of the complement system (see **Table 1** and **Figure 2** for an overview of clinical studies listed on clinicaltrials.gov). The pioneering drug in the field is eculizumab, a monoclonal antibody C5 inhibitor that was approved by the U.S. Food and Drug Administration (FDA) for treatment of the rare hemolytic disease paroxysmal nocturnal hematuria (PNH) in 2007, which represented a major breakthrough in this previously untreatable disease (15). The successful complement-targeting therapy has subsequently been explored in other diseases and has encouraged many companies to develop their own complement modulators, on one hand to offer therapies with improved properties compared to eculizumab, but also to target other diseases with a high unmet medical need (16).

**Table 1:** Clinical studies of terminal complement pathway inhibitors, as disclosed on clinicaltrials.gov (for eculizumab and ravulizumab, only studies with an active (not recruiting), recruiting, or not yet recruiting status are listed).

Trial Phase	NCT	Indication	Status	Estimated completion
Eculizumab, C5-targeting antibody (only active trials are listed)				
NA^1^	NCT04079257	PNH	Not yet recruiting	January 2021
2/3 3	NCT04155424 NCT02003144	Neuromyelitis Optica Spectrum Disorder	Recruiting Active, not recruiting	December 2024 June 2020
1	NCT04103489	HELLP Syndrome	Not yet recruiting	January 2023
NA^2^	NCT03950804	CHAPLE disease	Recruiting	June 2020
2	NCT03518203	aHUS-associated Multiple Organ Dysfunction Syndrome in Hematopoietic Stem Cell Transplant Recipients	Recruiting	December 2023
NA^3^	NCT03574506	Pregnancy-associated aHUS	Active, not recruiting	November 2020
4	NCT02013037	Antibody-mediated Rejection (cardiac transplantation)	Active, not recruiting	December 2019
3	NCT03759366	Refractory GMG	Recruiting	July 2025
2	NCT04346797	COVID-19	Recruiting	December 2020
2	NCT01029587	Renal Transplantation in Patients with History of Catastrophic Antiphospholipid Antibody Syndrome	Active, not recruiting	July 2020
2	NCT02029378	Guillain-Barre Syndrome Study	Unknown^4^	March 16
1	NCT03468140	Reduce Preservation Injury in Human Macrosteatotic Liver Transplantation	Recruiting	December 2020
Ravulizumab, C5-targeting antibody (only active trials are listed)				
4 3 3 3 1/2 2	NCT04320602 NCT02946463 NCT03056040 NCT03406507 NCT02598583 NCT02605993	PNH	Not yet recruiting Active, not recruiting Active, not recruiting Recruiting Active, not recruiting Active, not recruiting	February 2022 January 2023 March 2021 August 2022 June 2021 March 2022
3	NCT04201262	Neuromyelitis Optica Spectrum Disorder	Recruiting	July 2024
3	NCT03920293	GMG	Recruiting	December 2023
3	NCT04248465	ALS	Recruiting	October 2024
3 3	NCT04543591 NCT04557735	Thrombotic Microangiopathy Thrombotic Microangiopathy in children	Not yet recruiting Not yet recruiting	February 2024 February 2024
3 3 4	NCT04369469 NCT04570397 NCT04390464	COVID-19	Recruiting Not yet recruiting Recruiting	February 2021 November 2021 May 2022
3 3	NCT02949128 NCT03131219	aHUS aHUS in children	Active, not recruiting Active, not recruiting	July 2023 January 2025
2	NCT04564339	Lupus Nephritis or IgAN	Not yet recruiting	July 2023
Pozelimab, C5-targeting antibody				
2 3	NCT03946748 NCT04162470	PNH	Active, not recruiting Enrolling by invitation	May 2021 August 2023
2/3	NCT04209634	CHAPLE disease	Recruiting	July 2023
1 1	NCT04491838 NCT04601844	Healthy subjects Healthy subjects (in combination with Cemdisiran)	Not yet recruiting Not yet recruiting	March 2021 July 2021
Tesidolumab, C5-targeting antibody				
2	NCT02534909	PNH	Active, not recruiting	February 2022
1	NCT02878616	End-stage Renal Disease Patients Awaiting Kidney Transplant	Completed	July 2017
2	NCT02515942	Geographic atrophy	Completed	December 2017
1 2 2 2	NCT01255462 NCT01527500 NCT01624636 NCT01535950	AMD	Completed Completed Terminated Completed	September 2011 June 2015 May 2013 July 2013
2	NCT01526889	Active Non-infectious Intermediate-, posterior- or panuveitis	Completed	August 2017
2	NCT02763644	Transplant-associated Microangiopathy	Terminated^5^	June 2017
Crovalimab, C5-targeting antibody				
1/2	NCT03157635	PNH	Active, not recruiting	July 2025
3	NCT04434092	PNH	Not yet recruiting	October 2024
3	NCT04432584	PNH	Not yet recruiting	October 2024
ABP959, C5-targeting antibody (eculizumab biosimilar)				
3	NCT03818607	PNH	Recruiting	March 2022
SB12 C5-targeting antibody (eculizumab biosimilar)				
3	NCT04058158	PNH	Recruiting	October 2021
BCD-148, C5-targeting antibody (eculizumab biosimilar)				
3	NCT04060264	PNH	Recruiting	November 2020
Nomacopan, C5-targeting protein				
3 2 2	NCT03588026 NCT03427060 NCT02591862	PNH	Recruiting Recruiting Completed	January 2020 December 2019 March 2018
1/2	NCT04037891	Atopic Keratoconjunctivitis	Recruiting	February 2020
3	NCT03829449	aHUS/PNH (safety and efficacy surveillance study)	Recruiting	June 2025
2	NCT04035733	Bullous pemphigoid	Recruiting	March 2020
Zilucoplan, C5-targeting peptide				
2 2 2	NCT03078582 NCT03030183 NCT03225287	PNH	Completed Completed Active, not recruiting	March 2018 March 2018 January 2020
2	NCT04025632	Immune-Mediated Necrotizing Myopathy	Recruiting	November 2022
2 3 3	NCT03315130 NCT04115293 NCT04225871	GMG	Active, not recruiting Recruiting Recruiting	April 2020 April 2021 December 2022
2	NCT04382755	COVID-19	Recruiting	September 2021
2/3	NCT04297683	ALS	Recruiting	September 2021
Cemdisiran, C5-targeting oligonucleotide				
1/2	NCT02352493	PNH	Completed	August 2017
2 2	NCT03303313 NCT03999840	aHUS	Withdrawn Not yet recruiting	September 2018 February 2023
2	NCT03841448	IgAN	Recruiting	February 2023
1	NCT04601844	Healthy subjects (in combination with pozelimab)	Not yet recruiting	July 2021
Avacincaptad pegol, C5-targeting oligonucleotide				
2	NCT03362190	AMD	Completed	October 2018
2	NCT02686658	Geographic atrophy secondary to AMD	Completed	June 2020
2 2	NCT02397954 NCT03374670	IPCV IPCV	Completed Withdrawn^6^	October 2015 December 2019
2	NCT03364153	Autosomal recessive Stargardt disease	Recruiting	September 2020
3	NCT04435366	Geographic atrophy, macular degeneration	Recruiting	June 2023
ALXN1720, C5-targeting minibody				
1	Undisclosed^7^	Healthy subjects	Active, not recruiting	Initiated end of 2019
ALXN1007, C5a-targeting antibody				
2	NCT02128269	Antiphospholipid syndrome	Terminated^8^	June 2016
2	NCT02245412	Gastrointestinal Graft-Versus-Host Disease	Terminated^9^	February 2017
1 1	NCT01454986 NCT01883544	Healthy subjects	Completed Completed	October 2013 January 2014
IFX-1, C5a-targeting antibody				
2	NCT02246595	Sepsis (Septic Organ Dysfunction)	Completed	December 2015
2/3	NCT04333420	COVID-19	Recruiting	December 2020
2	NCT03971643	Pyoderma gangrenosum	Recruiting	April 2021
2	NCT02866825	Complex Cardiac Surgery	Completed	January 2017
2 2	NCT03895801 NCT03712345	AAV (Granulomatosis With Polyangiitis and Microscopic Polyangiitis)	Recruiting Active, not recruiting	July 2021 April 2021
2 2	NCT03001622 NCT03487276	Hidradenitis Suppurativa	Completed Completed	July 2017 January 2020
1	NCT01319903	Healthy subjects	Completed	October 2011
Avacopan, C5aR1-targeting small molecule				
2 2 3	NCT02222155 NCT01363388 NCT02994927	AAV	Completed Completed Completed	September 2016 January 2016 November 2019
2	NCT02464891	aHUS	Terminated^10^	July 2017
2	NCT02384317	IgAN	Completed	December 2016
2	NCT03852472	Hidradenitis Suppurativa	Active, not recruiting	December 2020
2	NCT03301467	C3 glomerulopathy	Recruiting	May 2021
Avdoralimab, C5aR1-targeting antibody				
2	NCT04563923	Bullous Pemphigoid	Not yet recruiting	December 2021
2	NCT04333914	COVID-19 Infection in Patients with Advanced or Metastatic Hematological or Solid Tumor	Suspended	August 2020
2	NCT04371367	COVID-19	Recruiting	October 2020
1	NCT03665129	Advanced Solid Tumors	Recruiting	June 2021
HMR59 (AAVCAGsCD59), MAC-targeting gene therapy				
1 1 2	NCT03144999 NCT03585556 NCT04358471	Dry AMD Wet AMD Dry AMD with geographic atrophy	Completed Active, not recruiting Not yet recruiting	December 2019 February 2021 September 2023

^1^Observational PK/PD study to develop a PK/PD-model for tailored treatment approach; ^2^transcriptome and Metabolic Analyses in CHAPLE patients (with and without eculizumab treatment); ^3^retrospective analysis of case series; ^4^status indicated as unknown by clinicaltrials.gov since no update since September 2014; ^5^terminated due to low confidence in clinical benefit; ^6^ terminated before any patient enrolled for portfolio reasons; ^7^disclosed in company press releases only; ^8^screening terminated due to slow enrollment; ^9^terminated at sponsor’s discretion, not due to safety concerns; ^10^terminated after enrollment of 6 out of 10 patients since study goals were accomplished.

AAV, antineutrophil cytoplasmic antibody-associated vasculitis; aHUS, atypical hemolytic uremic syndrome; ALS, amyotrophic lateral sclerosis; AMD, age-related macular degeneration; CHAPLE, complement hyperactivation, angiopathic thrombosis, protein losing enteropathy; COVID-19, coronavirus disease 2019; GMG, generalized myasthenia gravis; HELLP, hemolysis, elevated liver enzyme, low platelet; IgAN, IgA nephropathy; IPCV, idiopathic polypoidal choroidal vasculopathy; PD, pharmacodynamics; PK, pharmacokinetics; PNH, paroxysmal nocturnal hemoglobinuria.

Besides a differentiation from state-of-the-art therapy in target, epitope, or indication, physicochemical and pharmacokinetic (PK) properties could be a potential advantage of a new compound resulting in a more convenient administration route and regimen for patients with a life-long disease. In addition, to provide an example of the versatility of complement inhibitors and in light of the outbreak of the COVID-19 pandemic, implications of complement overreaction to infectious diseases are summarized, including the potential of complement therapies in COVID-19 (17, 18).

## Complement Cascade

The functions of the complement system are executed through a complex enzymatic cascade of more than 30 activating, amplifying, or regulating proteins or effectors (**Figure 1**) (19, 20). In brief, the complement is activated by three major pathways. The classical pathway is triggered mainly by antibody-antigen complexes, but also viral envelopes, cell envelopes of Gram-negative bacteria, C-reactive protein, or apoptotic cells. Specific moieties of these endo- or exogenous triggers are bound by the multimeric pattern-recognition molecule C1q, which recruits and activates serine proteases C1r and C1s. These, in turn, cleave complement proteins C2 and C4 into two fragments each. While C4a acts as anaphylatoxin inducing mast cell degranulation, C2b is suggested to increase vascular permeability (21). C4b and C2a form the C3 convertase, which splits C3 into C3a and C3b (22, 23).

Through the lectin pathway, the complement cascade is initiated by recognition of surface patterns on bacterial and fungal pathogens, as well as certain viruses. Thereby, mannose-binding lectins (MBLs) and ficolins are adopting the role of C1q to bind mannose residues on bacterial surfaces, while the proteolytic activity of C1r and C1s is covered by MBL-associated serine proteases (MASPs) to split C2 and C4 and generate the C3 convertase and, consequently, form C3a and C3b (23–25).

In contrast, the alternative pathway is constitutively active on a below-threshold level (tick-over), which requires active elimination of C3b deposition on endogenous cells and constant regulation (23). Therefore, native C3 is spontaneously hydrolyzed to C3(H_2_O), which recruits factor D, which cleaves factor B into the Bb fragment. Although C3(H_2_O) can associate with Bb to form a protein complex (C3(H_2_O)Bb) with similar function as the C3 convertase, the C3bBb complex is more efficient in cleaving C3 into C3a and C3b, indicating that the alternative pathway can also act as an amplification loop of complement effector function initiated by the classical and lectin pathways (19, 26).

Overall, the three pathways conclude in the formation of a C5 convertase, which is built through C3b binding to the existing C3 convertase complex. Therewith, C5 is cleaved into C5a and C5b, the terminal effector proteins of the complement system. Recently, a fourth way of complement activation has been introduced. Proteases otherwise not involved in the complement cascade, e.g., coagulation factors, have been found to directly cleave C3 and C5 (27, 28).

C5b recruits and binds complement proteins C6, C7, C8, and multiple units of C9 to form the membrane attack complex (MAC), a pore that inserts into lipid bilayer membranes of certain bacteria, parasites, enveloped viruses (29, 30), infected host cells, or, pathogenically, endogenous cells such as erythrocytes, to induce lysis (1, 19, 28). C5a, together with C3a, are potent anaphylatoxins modulating a plethora of cell types expressing G-protein coupled C5a or C3a receptors (C5aR1 and C3aR, respectively). In addition to increasing vasodilation and vascular permeability, inducing oxidative bursting of myeloid cells and, consequently, release of pro-inflammatory cytokines and chemokines, especially C5a has shown chemotactic properties for myeloid and certain lymphoid cells and to activate macrophages. The anaphylatoxins also interact with the coagulation system by modulating platelet response and the adaptive immune system by modulating cytokine synthesis of B-cells and initiating and regulating T-cell activation, including crosstalk with T-cell and Toll-like receptors. Furthermore, C3a and C5a effects on cell proliferation and tissue regeneration have been observed (4, 19, 28, 31–36).

Apart from C5aR1, a second C5a receptor, C5aR2 (or C5L2), was discovered, which is expressed less abundantly than C5aR1. Although few reports exist of C5aR2 modulating C5aR1 activity or exacerbating inflammation in disease models, it was initially mainly described to elicit anti-inflammatory effects, either as decoy C5a receptor or by independently downregulating cytokine responses and signaling (8, 37–40).

With involvement in numerous other physiological systems throughout the body, the complement cascade requires sensitive regulation. Besides decoy receptors, such as C5aR2, membrane-bound proteins that allow for differentiation of host and foreign cells as well as fluid-phase based proteins and cofactors prevent excessive activation and tissue damage. Most prominent, also in the context of rare inflammatory kidney diseases, is complement factor H (CFH), which disturbs C3 convertase formation of the alternative pathway, accelerates its decay, and binds to complement factor I (CFI) to lyse C3b (41). Many of them are involved in further biological processes, which again highlights the large network of the complement system throughout the body (5, 42, 43). Deficiencies in the complement pathways are known to lead to increased susceptibility to bacterial infections, but deficiencies in complement regulators either induce a depletion of certain complement components or inappropriate inflammation (44). Consequently, the potential for aberrant or inadequately regulated complement activity paves the way for disease (7, 36, 45). As one of the key effector functions, C5a-induced C5aR1 activation, is involved in numerous kidney diseases, but also cardiac remodeling in hypertension (46, 47), psoriasis (48), rheumatoid arthritis (49), complications in sepsis (50), neuropathic pain (51), or hidradenitis suppurativa (52). During the recent outbreak of SARS-CoV-2, the potential protective role of C5aR1 blockage in COVID-19-induced cytokine release and endothelial injury is being discussed (17).

## Inflammatory Kidney Diseases With Complement Involvement

The kidney is considered to be particularly vulnerable to damage caused by complement dysfunction (10, 53). Although the precise reason thereof is not completely understood, several factors might collude. While the majority of complement components is synthesized in the liver (90%), the kidney is one of the largest extrahepatic production sites (54–56). Due to the kidney’s predominant role in hemofiltration, the organ is prone to deposition of circulating immune complexes, which triggers inflammation and, subsequently, infiltration of immune cells (57). Its function in filtration and its continuous direct contact with antigens (23, 58) and a lower baseline abundance of complement regulators (59) is also thought to play a role. Overall, a significant body of evidence demonstrates that aberrant complement activation is involved in a number of kidney diseases such as lupus nephritis (60), immunoglobulin A nephropathy (IgAN) (5, 14, 61, 62), atypical hemolytic uremic syndrome (aHUS) (63, 64), C3 glomerulopathy (65), and antineutrophil cytoplasmic antibody (ANCA)-associated vasculopathy (AAV) (66).

### Lupus Nephritis

Renal involvement in the chronic autoimmune disease systemic lupus erythematosus (SLE), manifesting as lupus nephritis, is common, with a prevalence of up to 48% of SLE patients (67), and is usually associated with poor outcome. Pathological features consist of glomerulonephritis and glomerular crescents, infiltration of inflammatory cells in the kidney’s epithelium, podocyte injury, immune complex deposition on mesangial cells, and vascular and tubulointerstitial lesions (68). The interrelationships between complement and SLE are complex and the pathogenesis of lupus nephritis is not fully elucidated. Genetic deficiency of C1q is a strong predictor for developing SLE. Moreover, anti-C1q autoantibodies and corresponding immune complexes relate to renal involvement, highlighting the protective function of the classical pathway in clearing immune complexes. However, once the disease is triggered and immune complexes are insufficiently cleared, their deposition in tissue leads to local complement activation, mainly driven by terminal complement components, indicated by decreased systemic levels of C3 and C4, suggesting high inflammatory activity and tissue damage (69–73). Furthermore, complement has been shown to contribute to the disease by increased C3aR and C5aR activity (74, 75), and elevated C5a levels (76). State-of-the-art clinical management of lupus nephritis consists of nonselective immunosuppressive treatment with corticosteroids (68). In addition, several selective immunosuppressors are currently in clinical development, e.g., B-cell activating factor inhibitors or anti-CD20, -CD40L, and -type 1 interferon monoclonal antibodies (77), which act through modes of action different from complement inhibitors and thereby could provide synergistic benefit.

### Immunoglobulin A Nephropathy

Although IgAN with a yearly incidence of 0.2 to 2.8 per 100,000 as the most common cause of glomerulonephritis (78), it is an orphan disease with a prevalence of 4 in 10,000 people by the European Medicines Agency (79). The disease is caused by abnormally glycosylated IgA, recognized as autoantigens by properly functioning IgA and IgG. Subsequently formed immunocomplexes deposit in the glomerular mesangium and locally activate the complement cascade, which eventually leads to podocyte and glomerular injury, fibrosis, and ESRD in up to 40% of patients (80). Several complement proteins, most frequently C3 and alternative-pathway regulators, have been found to co-deposit with IgA deposits, contributing to renal injury. In addition, also C4, together with MBL and ficolins, are detected in mesangial deposits, whereas C1q is rarely found, suggesting that the lectin rather than the classical pathway plays a role in IgAN (81, 82). Genome-wide association studies indicated a link between rare genetic variants of CFH-related (CFHR) protein 5 and IgAN susceptibility, although their contribution to the disease is unknown (83). An IgAN induction protocol, in which mice are immunized to Sendai virus to develop an IgA-mediated immune response and corresponding immune complex deposition (84), was applied in C3aR- and C5aR-deficient mice. Anaphylatoxin deficiency resulted in decreased renal IgA and C3 deposition, less secretion of proinflammatory cytokines, and a protective effect on the kidney as indicated by reduced proteinuria (85), while urinary and renal C3a and C5a levels correlated with disease severity (14). Besides diet and lifestyle modifications, blockage of the renin-angiotensin system (RAS) has been shown to significantly reduce the decline in renal function (86). Nonselective immunosuppression, particularly corticosteroid therapy, is used as second-line management, however, improved long-term outcome could not be demonstrated in the majority of patients, while safety and tolerability are compromised with the use of corticosteroids (87).

### Atypical Hemolytic Uremic Syndrome

In the majority of patients diagnosed before 16 years of age, aHUS is a rare disease that presents with microangiopathic hemolytic anemia, thrombocytopenia, and acute renal failure (64) with a prevalence of 2.2–9.4 per 1,000,000 (88). A number of genetic deficiencies of complement components lead to impaired regulation of the alternative pathway, either through loss-of-function mutations of regulating proteins, i.e., CFH or CFI, or through gain-of-function mutations of C3, leading to reduced affinity of CFH, or complement factor B, forming a hyperactive C3 convertase, thereby overwhelming regulatory capacity (89). Moreover, certain genetic variants of CFH induce generation of autoantibodies that neutralize CFH and prevent complement regulation (90). Mutated CFH and CFHR hybrid proteins are unable to protect endogenous cells from MAC-induced phenotype alteration of endothelial cells, favoring platelet aggregation (91). C3a- and, most excessively, C5a-mediated inflammation and myeloid cell immigration lead to a prothrombotic state, especially in the endothelium, since the fenestrated structure favors exposure to circulating complement components. Eventually, endothelial swelling manifests in collapsed capillaries, which is fatal in 20%–80% of cases, depending on the mutated complement regulation gene (92, 93). Treatment options consist of plasma exchange combined with nonselective immunosuppression with the goal to replace mutant CFH, however, therapy benefit was only temporary and not as successful in patients with CFI or C3 mutations. After kidney transplantation, disease recurrence rates were as high as 50% (92). More recently, eculizumab was shown efficacious in aHUS by interrupting further uncontrolled complement activation through endothelial injury. This resulted in normalized platelet counts and hematologic values, no thrombotic microangiopathic events during treatment, and improved kidney function reflected, among others, as being dialysis-free in a significantly larger proportion of patients compared to previous treatment options (94, 95).

### C3 Glomerulopathy

C3 glomerulopathy consists of several subforms that are characterized by continuous local complement activity, resulting in C3 deposition in renal tissue, without co-deposition of immunoglobulin, with clinical signs of glomerulonephritis, i.e., hematuria, hypertension, and acute and chronic kidney injury. The disease is extremely rare, with a prevalence of 0.1–5 per 1,000,000, and up to 70% of patients progress to ESRD within 10 years after diagnosis (96). Two main subtypes within the term of membranoproliferative glomerulonephritis (MPGN) are distinguished: C3 glomerulonephritis (C3GN) is identified by subendothelial and mesangial depositions, while in dense-deposit disease (DDD), the deposits are found intramembranous and more electron-dense (13). In most patients, the alternative pathway is dysregulated through C3 convertase stabilization by autoantibodies such as C3 nephritic factor, but also C5 convertase binding autoantibodies are reported. Both lead to chronic and unregulated complement activation in the fluid phase. Genetic deficiencies of CFH prevent its regulatory function, either by defective protein processing or lacking secretion. In addition, a variety of deletions, duplications, and chromosomal rearrangements in 5 CFHR protein genes, which are located in the same gene cluster, are known to disturb dimerization of CFHR proteins, thereby disturbing CFHR protein equilibrium and CFH activity in the fluid phase or are associated with CFH autoantibodies. Moreover, mutated C3 forms are reported that can generate C3 convertases resistant to CFH or CFI-mediated decay (96–100). Besides these complement-mediated MPGN forms, immune complex-mediated MPGN has been differentiated, in which the classical pathway is the driver of disease and immune complexes are found to deposit in glomeruli (101). Patients are mainly treated with RAS blockers, since no therapy is specifically approved for C3 glomerulopathy. Plasma exchange and nonspecific immunosuppression have also been assessed in several patients, however, results remain controversial. C5-targeting eculizumab improved the clinical response in a subset of patients only, which might be due to the antibody not improving the C3 dysregulation (96), and C5 convertase overactivity being observed mainly in C3GN compared to DDD (13). Although C3 is the main driver of the disease, C5aR blockage is suggested to add substantial benefit for C3 glomerulopathy patients, which is supported by positive findings with eculizumab in prospective clinical trials (99, 100, 102, 103).

### Antineutrophil Cytoplasmic Antibody-Associated Vasculopathy

AAV includes three different conditions, i.e., granulomatosis with polyangiitis, microscopic polyangiitis, and eosinophilic granulomatosis with polyangiitis. Annual incidence rates are between 0.5–14.4 per 1,000,000 in Europe and 5-year survival is around 75% (104). The exact etiology remains unresolved, but several genetic mutations and epigenetic modifications have been associated with increased susceptibility to an increased responsiveness of the immune system and to develop ANCA. Certain infections, drugs, or airborne particles can trigger AAV by inducing an initially normal immune response, in which C5a–C5aR1 binding and pro-inflammatory cytokines from macrophages prime neutrophils to express bacteria-degrading proteins, such as myeloperoxidase (MPO) or proteinase 3 (PR3), and form neutrophil extracellular traps (NETs). In AAV patients, NET degradation is decreased and, after prolonged exposure, tolerance to self-antigens on neutrophil surfaces wanes. Consequently, ANCAs bind MPO and PR3 and further activate neutrophils. This excessive production of cytokines and reactive oxygen species induces vascular injury and necrosis in a vicious circle (105). AAV is treated with high-dose corticosteroids with subsequent tapering, in combination with cyclophosphamide or, due to cyclophosphamide toxicity, rituximab or mycophenolate mofetil (106). However, an increasing number of complement-targeting therapies have been tested in AAV in recent years (107). A first step in this direction was taken with avacopan, a small molecule C5aR1 antagonist that induces vasculitis remission while replacing corticosteroids (see Avacopan (CCX-168)) (108, 109).

## Terminal Complement Pathway-Targeting Treatments of Inflammatory Kidney Diseases

As a summary, treatment options in above-mentioned diseases mainly consisted of strong, nonspecific immunosuppressive agents, including high-dose corticosteroids (68, 86). Though dampening immune system-related symptoms, corticosteroids bear considerable treatment-related morbidities. The consequences are adverse events (AEs) that increase with treatment of longer duration, including risk of infection, weight gain, new onset of diabetes, hypertension, and/or osteoporosis (12, 110). In the STOP-IgAN trial, safety and efficacy in IgAN patients on corticosteroids (and nonspecific immunosuppressive agents) compared to supportive care were assessed for 3 years. Eventually, immunosuppression by corticosteroids did not yield any substantial clinical benefits, while more infections and comorbidities were reported (87). Since complement has been shown to be involved in these diseases, compounds that selectively target complement components might provide a valuable treatment alternative.

### Eculizumab (Soliris^®^)

As a first breakthrough in complement-targeting therapy, the recombinant humanized monoclonal antibody eculizumab received accelerated approval as first-in-class C5 inhibitor in PNH in 2007 (111). PNH is a rare hemolytic disease, in which mutations in the genes for hematopoietic stem cells lead to a lack of complement regulatory protein expression on the surface of erythrocytes. Consequently, these are attacked by a constantly activated alternative complement pathway, inducing vascular hemolysis and thrombosis (45). Prior to eculizumab approval only supportive measures were available to increase resynthesis of erythrocytes and prognosis was poor, whereas eculizumab successfully and over a prolonged time frame prevents erythrocyte lysis (112, 113). The selective binding of eculizumab to C5 prevents C5 cleavage and subsequent formation of proinflammatory C5a and cell lysing MAC, while preserving upstream C3a- and C3b-mediated effects (114). After several promising off-label uses of eculizumab in aHUS (115–117), the drug was approved for aHUS based on 5 single-arm studies in 2011 (94, 118, 119). Subsequently, it received approval for the neuromuscular disease generalized myasthenia gravis (120) in 2017 (121) and the eye disorder neuromyelitis optica (122) in 2019 (123). Currently, eculizumab is assessed or used off-label in a range of other indications (124), such as cold agglutinin disease (125), membranoproliferative glomerulonephritis (126), antibody-mediated rejection of transplants (127), and, as mentioned above, C3 glomerulopathy (115). In adult patients, eculizumab is administered as 35 min intravenous infusion of 900 and 1,200 mg in PNH and aHUS, respectively, in weekly intervals in the first four weeks, followed by maintenance doses every 2 weeks (118).

The success of eculizumab clinically validated the inhibition of complement and paved the way for further therapeutic agents against complement components. However, eculizumab’s route of administration and PK properties require a bimonthly infusion and, therefore, a significant burden for patients (6). Also, due to a large inter-individual variability of PK parameters among patients, several studies assessed therapeutic drug monitoring and tailored dosing regimens to maintain efficient complement suppression while avoiding excessive trough concentrations (121, 128, 129). In addition, at the time of market entry, the antibody was the most expensive therapy globally, which fueled many debates on the ethics of cost-effectiveness (130, 131). Although Alexion, the drug manufacturer, secured a patent extension for eculizumab to 2027 (Alexion press release, 15 August 2017), other companies are trying to conquer their share of the PNH market through various biosimilars that are clinically assessed, such as ABP 959 or BCD-148 (132, 133), or already received approval in certain countries, such as Elizaria^®^ (134) and SB12 (Samsung Bioepis press release, 10 January 2020). Further, eculizumab, as a C5-neutralizing antibody, inherently blocks the MAC formation, which increases the susceptibility to bacterial infections. An increased risk of infections, mainly with *Neisseria meningitidis*, but also case reports of meningococcal or *P. aeruginosa* infections (135, 136), has been observed, requiring patients to be vaccinated (137–139). In addition, a complete suppression of complement activity has not been observed in all eculizumab-treated patients, resulting in breakthrough hemolysis either at the end of the eculizumab dosing interval or, irrespective of the antibody’s plasma concentration, during pathogenic infection provoking strong complement activity (140). Also, polymorphisms of the C5 gene have been identified that lead to C5 variants with an epitope that does not allow eculizumab binding and subsequent C5 blockade (141).

Therefore, a variety of novel therapies is currently being developed against complement-mediated diseases, filling the legacy that eculizumab provided and aiming to overcome the shortcomings of eculizumab (11, 119, 123, 142). An overview of therapeutics targeting the terminal complement pathway that are in clinical development and their intended indications is provided (**Figure 2**).

### Ravulizumab (ALXN1210; Ultomiris^®^)

As a follow-up treatment to eculizumab, Alexion developed ravulizumab to target the same epitope of C5, but extended antibody recycling through enhanced affinity to the neonatal Fc receptor, which prolonged the terminal half-life by 4-fold and, consequently, the infusion intervals from 2 to 8 weeks (143). However, C5 affinity is 17-fold lower compared to eculizumab (144). Nonetheless, non-inferiority compared with eculizumab, including safe and effective switch from eculizumab to ravulizumab therapy, was established in two Phase 3 trials in PNH (145, 146). In addition, fewer events of breakthrough hemolysis were reported for the follow-up antibody, indicating a more stable C5 inhibition and improved patient safety (147). After a successful prospective open-label Phase 3 trial, ravulizumab was approved also in aHUS (148). On 14 January 2020, Alexion announced that a Phase 3 trial in amyotrophic lateral sclerosis is planned to be initiated, thereby expanding the scope of terminal complement inhibitors to neurological diseases (Alexion press release, 14 January 2020). The FDA label foresees maintenance doses of up to 3,600 mg (depending on body weight) ravulizumab administered intravenously up to 2 h every 8 weeks, starting 2 weeks after a loading dose (149). However, the risk of infections by encapsulated bacteria, inherent to the inhibition of MAC, despite patient vaccination also exists with ravulizumab (150). Nonetheless, ravulizumab is planned to be explored in further indications, since clinical trials in thrombotic microangiopathy, lupus nephritis, and IgAN have recently been set up (151–153).

### ALXN1720

Further to eculizumab and ravulizumab, Alexion is developing ALXN1720, a bi-specific anti-C5 mini-body that binds human C5 and prevents its activation, currently assessed in a Phase 1 healthy subject study (Alexion press release, 6 May 2020). However, no indications or further details have been released.

### Pozelimab (REGN3918)

This fully humanized antibody against C5 has been shown to suppress C5 levels and hemolysis in human serum and mice models in a more potent manner compared to eculizumab and ravulizumab (154, 155). In a Phase 1 study in healthy subjects, pozelimab also showed 70% bioavailability after subcutaneous administration, allowing for more versatile administration options, such as weekly subcutaneous 400 mg administrations after a 15 mg/kg intravenous loading dose (156). At the end of 2019, positive results of pozelimab in a Phase 2 study in PNH were announced (Regeneron press release, 5 December 2019). The same treatment regimen is also being applied in a recently started Phase 2/3 study in patients with CD55-deficient protein-losing enteropathy, an intestinal inflammatory disease with complement contribution (157, 158).

### Tesidolumab (LFG316; NOV-4)

This human monoclonal IgG1-antibody against C5 was originally developed against the eye disease age-related macular degeneration (AMD), one of the major causes of blindness in the world, in which C5a and C5b are deposited in retinal cells and Bruch’s membrane, causing inflammation and cell lysis (159). However, effectiveness in AMD could not be established (159). Trials with tesidolumab in transplantation-associated microangiopathy and several forms of uveitis have only shown limited clinical benefit (160, 161). So far, tesidolumab has been administered as intravitreal injection every 28 days (162). Novartis is currently assessing intravenously administered tesidolumab in a Phase 2 study in PNH, however, without indicating the treatment regimen (163). Interestingly, in ESRD patients awaiting kidney transplantation, drug-drug interactions between high-dose intravenous immunoglobulin, a common therapy to reduce antibody-mediated rejection after transplantation, and intravenous tesidolumab were reported, though the latter was more rapidly eliminated (164).

### Crovalimab (SKY59; RG6107; RO7112689)

Similar to ravulizumab, crovalimab was engineered to employ pH-dependent antibody recycling for an increased half-life (165, 166). Crovalimab binds to C5 on a different epitope than eculizumab, which would enable C5-neutralization in patients carrying certain genetic polymorphisms that prevent eculizumab binding (167). In addition, crovalimab was also found to bind C5b, thereby inhibiting MAC formation, which could prevent cell lysis in cases where C5 is cleaved by proteases of the coagulation system (168). Pharmacokinetically, crovalimab can be administered subcutaneously with a bioavailability of 90%, which allows for self-administration and reduces treatment burden (169). In a combined Phase 1/2 study in PNH patients, efficacy and tolerability of subcutaneous dosing regimens of weekly up to once every 4 weeks were assessed and switching from eculizumab to crovalimab was considered safe (170).

Monoclonal antibodies excel through their target specificity and allow for targeting complex protein-protein interactions or conformational changes that remained inaccessible for small molecules for a long time, while only few safety issues owing to off-target effects are expected (171). However, high plasma concentrations are usually necessary, which require high-dose bolus injections that can trigger hypersensitivity and infusion reactions, such as dyspnea, nausea, headache, or skin reactions (172). Neutralizing immune responses such as anti-drug antibodies may develop which reduce efficiency, influence the PK properties and toxicity profile, and corresponding immune complexes can induce anaphylaxis or allergic reactions (173). In addition, mechanism-dependent toxicities cannot be avoided, in which case a longer half-life compared to oral treatments is a disadvantage (174). Administration routes and treatment regimens also present a downside. Even though larger antibodies usually present longer half-lives, which would decrease treatment frequency and facilitate compliance, their ability to penetrate into tissue is limited, which narrows down the range of accessible targets. Subcutaneous administration is also restricted by the size of the molecule (173). In diseases with incisive implications related to survival and quality of life, stationary treatment is common and, therefore, parenteral administration is not considered a limitation. Nonetheless, an orally available treatment option would present more flexibility and convenience in a chronic disease (119). Furthermore, monoclonal antibodies present a significant financial burden in long-term therapies (115), as their production based on cell lines and subsequent purification processes remains tedious and expensive (174). As for any biological macromolecule, a properly folded tertiary structure of a monoclonal antibody is crucial for its activity, however, these structures can be prone to denaturation under stress conditions, which further complicates formulation efforts (173).

In a first step to decrease the molecular weight of a potential complement inhibitor, while preserving the target specificity, proteins, peptides, and oligonucleotides are bridging the way to small molecules and expanding treatment options in complement-mediated diseases (6).

### Nomacopan (Coversin; rVA576)

Nomacopan is a recombinant small protein derived from a tick C5 inhibitor with a binding epitope opposite of eculizumab’s (140, 175). Besides C5, it has also been shown to inhibit leukotriene B4, a contributor to skin inflammation. Nomacopan was successfully tested in a mouse model of the skin disease bullous pemphigoid, which is mediated by C5a and leukotriene B4 (176). On 1 May 2020, Akari Pharmaceuticals announced positive results from a Phase 2 study in pemphigoid diseases (Akari press release, 1 May 2020). Recently, case reports of successful inhibition of complement-mediated hemolysis by nomacopan in a PNH patient non-responsive to eculizumab (177) and a thrombotic microangiopathy patient (178) were reported. In May 2018 and July 2018, a Phase 2 study in PNH patients with resistance to eculizumab and a Phase 3 study in PNH were initiated, respectively (179–181). According to a press release from November 2018, nomacopan is also assessed in aHUS and post-transplant thrombotic microangiopathy patients (Akari press release, 20 November 2018). Besides applications in kidney and hematologic diseases, nomacopan was efficacious in preclinical models of sepsis (182), while it also showed preliminary efficacy in atopic keratoconjunctivitis patients (Akari press release, 14 October 2019). The diverse potential of nomacopan currently is only dulled by its unfavorable PK. Due to the short half-life (10 h), daily subcutaneous injections would be necessary. A modification of the protein with an N-terminal fusion tag of 600 amino acids (PASylation) could extend the half-life and allow for once weekly dosing, without compromising C5 inhibition potency (183, 184).

### Cemdisiran (ALN-CC5)

Cemdisiran is the first compound applying the concept of RNA interference to complement inhibition. Subcutaneous administration of small interfering RNA sequences matching C5 mRNA, conjugated with N-acetylgalactosamine for targeted delivery to hepatocytes, silenced C5 synthesis and reduced hemolytic activity in monkeys by 80% in weekly or twice weekly treatment regimens (185). Weekly dosing in PNH patients in a Phase 1/2 study decreased serum C5 levels by 98% and allowed for a subsequent reintroduction of eculizumab in a sparing dosing regimen of once monthly (Alnylam press release, 5 December 2016). However, low levels of residual hemolysis were observed due to remaining extrahepatic C5 synthesis and even after addition of eculizumab (123, 186). As a potential option to prevent residual hemolysis, cemdisiran was announced to be assessed as combination therapy with pozelimab (Regeneron press release, 8 April 2019) (187). In addition, cemdisiran is being investigated in IgAN (185) and aHUS patients (Alnylam press release, 26 September 2016) at 600 mg once every 4 weeks with option to decrease dosing frequency. In the latter, an initial Phase 2 study was terminated due to lack of enrollment (188), but efforts are continued in a new study evaluating the switch from eculizumab to the oligonucleotide in aHUS patients (189).

Even though C5 inhibition provided sweeping success in many complement-mediated diseases, the inherent inhibition of MAC formation requires vaccination of patients to *Neisseria meningitidis*. Additionally, increased predisposition to infections with other bacteria has been reported (137). To overcome this safety burden, direct inhibition of C5a or C5aR1 would allow for anti-inflammatory activity in diseases mediated by the anaphylatoxin without compromising MAC formation. In addition, inhibition of the C5a–C5aR1 axis has been found to alleviate *Neisseria*-induced sepsis in mice (190). Moreover, C5a generated through direct C5 cleavage by proteases other than C5 convertase can be neutralized with agents directly targeting the C5a-C5aR1 axis (191).

### ALXN1007

Not surprisingly, Alexion has applied its knowledge in complement-mediated disease to develop ALXN1007, a once or twice weekly intravenously administered monoclonal antibody targeting C5a that has been granted orphan drug designation for graft-versus-host disease by the FDA (Alexion press release, 19 October 2016). However, after promising results with a 77% complete response from the first cohort of weekly 10 mg administrations (192), the study was terminated early without stating the underlying reason, although not due to safety concerns. A possible explanation could be the overall disease response rate being lower at higher dose levels (193). In another Phase 2a study, ALXN1007 was assessed in antiphospholipid syndrome, which was terminated early as well, this time due to slow enrollment (194).

### IFX-1

The chimeric monoclonal antibody IFX-1 is directed against C5a without interfering with C5 cleavage, thereby leaving MAC formation intact (191). Initially assessed in sepsis and septic shock (195) and as single intravenous dose in complex cardiac surgery (196) without reporting any results, IFX-1 showed most promising efficacy in hidradenitis suppurativa. This is a chronic inflammatory skin disease presenting with painful and stigmatizing abscesses and significant comorbidities. Many links to immune dysregulation have been found, but the exact etiology remains unknown and, consequently, therapeutic options are limited (52, 197, 198). In a prospective open-label Phase 2a study, twice weekly followed by once weekly intravenous infusions of IFX-1 were well tolerated and improved hidradenitis suppurativa clinical response scores were reached by 83% of patients (199). In the following confirmatory placebo-controlled Phase 2b study, IFX-1 failed to meet the primary endpoint of a dose-dependent effect on the hidradenitis suppurativa clinical response rate after once or twice monthly administrations for 16 weeks (InflaRx press release, 5 June 2019). However, secondary endpoint, post-hoc, and long-term extension results suggested multiple efficacy signals (InflaRx press releases, 18 July and 6 November 2019). In addition, InflaRx reported preliminary positive results of an ongoing Phase 2a study in pyoderma gangraenosum, an autoinflammatory ulcerating skin disease without approved therapy (InflaRx press release, 26 February 2020). Besides skin disorders, IFX-1 is currently tested in systemic versions of AAV on top of standard of care (200) as well as replacement for corticosteroid therapy (201). In addition, as IFX-1 administered in African green monkeys infected with H7N9 influenza virus alleviated acute lung injury and systemic inflammation (202), an open-label Phase 2/3 study in COVID-19-infected severe pneumonia patients has been initiated (203).

### Avacopan (CCX168)

With avacopan, the first low molecular-weight orally available C5aR1 antagonist has reached clinical development. Avacopan potently and selectively inhibits C5a-mediated effector functions *in vitro*, as well as in mice glomerulonephritis (204) and monkey neutropenia models after daily oral administration (205). In a Phase 1 study in healthy subjects, single and multiple doses up to 100 mg and 50 mg twice daily, respectively, were well tolerated. The small molecule was rapidly absorbed after oral administration, reaching peak plasma concentrations after 1 to 2 h, but a similarly fast clearance from plasma in a biphasic manner was observed. At 12 h post-dose, plasma levels decreased to around 80% of peak plasma concentrations, although thereafter, the terminal elimination half-life was more than 120 h after multiple administrations above 10 mg (205). Consequently, a twice daily avacopan dosing regimen was selected for the Phase 2 studies in AAV. Since no C5aR1 antagonist had been administered to AAV patients before, the safety of twice daily 30 mg avacopan during corticosteroid therapy tapering, followed by 30 mg avacopan as corticosteroid replacement in a new cohort, both on top of background cyclophosphamide or rituximab therapy, was evaluated, before an efficacy endpoint was added in the Phase 2 CLEAR study. The avacopan arm presented a reduction in the Birmingham vasculitis score (BVAS) in a larger number of patients compared to the corticosteroid arm, while fewer AEs were reported (108). Subsequently, the Phase 2 CLASSIC study further characterized the safety profile of 10 and 30 mg avacopan in addition to standard of care (high-dose corticosteroids plus cyclophosphamide or rituximab) (109).

Recently, preliminary results of the Phase 3 study in AAV patients (206) became available, indicating that avacopan was non-inferior and superior after 26 and 52 weeks, respectively, compared to corticosteroids, in the BVAS-assessed vasculitis remission, while renal function and corticosteroid-induced toxicity were significantly improved under avacopan (207).

Besides being successfully developed in AAV and providing a valuable treatment alternative to corticosteroids, avacopan is being investigated in Phase 2 studies in several other indications. In an open-label pilot study in IgAN patients on full RAS blockade, twice daily avacopan administration led to a clinically meaningful decrease (50%) of the primary endpoint, urinary protein to creatinine ratio, after 12 weeks in 3 of 7 patients, while being well tolerated (208). Another open-label Phase 2 study of 15-day avacopan treatment in aHUS patients with ESRD was terminated after enrollment of 6 patients without disclosing any results (209). Furthermore, topline results from a placebo-controlled Phase 2 study in hidradenitis suppurativa were recently announced, indicating statistically significant improvements in severe cases compared to placebo (Chemocentryx press release, 28 October 2020). Another Phase 2 study in C3 glomerulopathy is currently ongoing (210), for which Chemocentryx was granted orphan drug designation by the EMA and the FDA (Chemocentryx press release, 23 May 2017).

## Terminal Complement Pathway Inhibitors in Other Indications

### Zilucoplan (RA101495)

The small macrocyclic peptide zilucoplan specifically and potently binds C5 at an epitope located on the C5b part, thereby also binding C5b fragments. Daily subcutaneous self-administrations of zilucoplan were efficacious in a Phase 2 study in generalized myasthenia gravis (211), after which a Phase 3 study was initiated at the end of 2019 (212). In addition, the peptide is assessed in immune-mediated necrotizing myopathy (213) and as part of a perpetual platform study to test several treatments in amyotrophic lateral sclerosis (214).

With the success in myasthenia gravis, Ra Pharmaceuticals, recently acquired by UCB Pharma (UCB press release, 2 April 2020), announced to postpone developing zilucoplan in PNH. Although preliminary positive results of the Phase 2 study are reported (215), residual hemolytic activity and breakthrough hemolysis was still observed under zilucoplan treatment (169). In addition, an extended release formulation is being developed to improve the treatment regimen (Ra Pharmaceuticals press release, 9 April 2019). Positive results in Phase 1b studies in renal indications are announced on their webpage, however, no further details are provided (216). On 11 May 2020, a prospective clinical study of zilucoplan in COVID-19 was initiated (217).

### Avacincaptad Pegol (ARC1905)

Similar to cemdisiran, avacincaptad pegol is a single-stranded oligonucleotide (also termed aptamer), but instead of silencing C5 protein synthesis, avacincaptad pegol directly binds and inhibits C5 (218). Aptamer technology is reported to have high target affinity and specificity without requiring expensive biosynthesis, which also allows for more flexibility in structural adaptations during *in vitro* selection compared to antibodies. Specific modifications introduced resistance to nuclease-dependent degradation, which increased the half-life of aptamers to several days (219). Avacincaptad pegol is applied as monthly intravitreal injections in the complement-mediated eye disorders Stargardt Macular Dystrophy, idiopathic polypoidal choroidal vasculopathy (IPCV), and geographic atrophy in AMD (159). In the latter, Iveric bio Inc announced statistically significant dose-dependent reduction in geographic atrophy growth with avacincaptad pegol in a Phase 2b study, meeting its primary endpoint (Iveric bio press release, 28 October 2019). Early 2020, the company launched a Phase 3 study in said eye disease (Iveric bio press release, 27 February 2020), for which fast track designation was received from the FDA (Iveric bio press release, 3 April 2020). Besides, results of a Phase 2b study in Stargardt Macular Dystrophy are expected mid 2020 (Iveric bio press release, 27 February 2020). Efforts in IPCV were terminated for portfolio reasons (220). Overall, avacincaptad pegol offers a well-tolerated treatment option in eye diseases with great unmet medical need, although the route of administration of monthly intravitreal injections still implies inconvenience, since most AEs in the Phase 2b study in AMD were due to the injection procedure (Iveric bio press release, 28 October 2019).

### Avdoralimab (IPH5401)

Avdoralimab, developed by Innate Pharma, underlines the versatility of the complement system. This fully human anti-C5aR1 antibody is being developed in advanced solid tumors, in which C5aR1 activity is suggested to suppress T cell and natural killer (NK) cell activity in tumor immune surveillance (221). In a Phase 1 study in patients with advanced solid tumors in combination with anti-PD-1 therapy, avdoralimab showed full C5aR1 blockage and early efficacy signals (222). On its webpage, the company disclosed the initiation of clinical trials of avdoralimab in chronic spontaneous urticaria and bullous pemphigoid (223). Furthermore, the effect of anti-C5aR1 activity in COVID-19 patients with severe pneumonia is currently being assessed in a Phase 2 study with avdoralimab (224).

### MAC Inhibitor HMR59 (AAVCAGsCD59)

Hemera Biosciences has taken a different approach to complement inhibition, although at the moment applied to AMD only. HMR59 is an intravitreally injected gene therapy that increases production of soluble recombinant CD59, a regulator of C9 of the MAC, in retina cells to protect them from MAC-induced cell lysis (225). The effect of a single injection of HMR59, which is expected to suffice for a life-long effect, was assessed over 18 months in an open-label Phase 1 study in AMD patients (226). Although no results are disclosed, the subsequent placebo-controlled Phase 2 study in AMD patients is estimated to start in June 2020 (227).

### Latest Emerging Indication for Complement Inhibitors: COVID-19

Besides inflammatory kidney, hematologic, neurologic, and eye diseases discussed above, there is evidence accumulating to suggest that complement is involved in SARS-CoV-2 pathology (18). Particularly, the concept of cytokine storms within the body damaging organs and tissue recently gained fresh attention with the outbreak of the COVID-19 pandemic (228, 229).

SARS-CoV-2 infects cells by endocytosis through binding to the angiotensin-converting enzyme 2 (ACE2), which is expressed on cells in the human upper airway and pneumocytes (230). In the incubation and initial (i.e., non-severe) phase, a speciﬁc adaptive immune response is required to control the virus and to preclude disease progression to the more severe phase (229), mainly consisting of cytotoxic T cells and NK cells (231). Overall, 80% of SARS-CoV-2 infected individuals present mild symptoms. In some patients, the absence of effective activation of T cells and NK cells leads to ineffective SARS-CoV-2 viral clearance and weak antibody production (230). The patient enters a severe phase and develops COVID-19 pneumonia, characterized by a destructive inﬂammatory response through myeloid cell (monocytes and macrophages) infiltration and, consequently, cytokine release syndrome, particularly in the lungs, as lung epithelium and endothelium are primary targets of SARS-CoV-2 (229).

In the context of influenza viral infection, the complement pathway is activated and in animal models, inhibition of C5a has been shown to be beneficial in improving disease outcome (202, 232, 233). Furthermore, for other species of the coronavirus family, complement contribution to disease starting from Day 1 post infection has been shown earlier (234). Inhibition of C5aR1 activity has been shown to alleviate disease severity and organ damage by reducing inflammation in MERS-CoV infections (235). A similar effect has been suggested in SARS-CoV (228, 234) and increased C5a levels have been found in COVID-19 patients (18). Consequently, several C5, C5a, and C5aR1 inhibitors are currently undergoing clinical studies in COVID-19, such as eculizumab, which has already showed preliminary efficacy (236), ravulizumab (237), zilucoplan (217), IFX-1 (203), and avdoralimab (224).

Current measures to contain the spread of SARS-CoV-2 include social and physical distancing to protect vulnerable populations, until a treatment or a vaccine is shown to be efficacious. In that sense, complement inhibitors could potentially reduce the severity of COVID-19-induced complications and the number of patients in intensive care or fatalities. Consequently, the impact on the health care system would be reduced, improving the overall disease burden on society, particularly since the long-term implications of severe COVID-19 cases and intubation are heavily discussed.

## Conclusion

Through the introduction of eculizumab to the market in 2007, the paradigm of complement inhibitors as a successful therapy in rare (auto)inflammatory diseases has been validated. Many of these debilitating disorders did not have an approved treatment available and either were connected with early mortality or inconvenient stationary therapy (plasma exchange, dialysis) (238). Otherwise, physicians were relying on strong nonspecific immunosuppression by cyclophosphamide, azathioprine, methotrexate, rituximab, and mycophenolate, for which the dose has to be carefully adjusted and monitored due to inherent toxicity (12). In addition, corticosteroids were abundantly used due to good accessibility, inexpensive treatment, and the possibility of oral therapy. However, corticosteroids bear well-known and significant comorbidities for long-term administration, such as an increased infection risk, diabetes, osteoporosis, weight gain, myopathy, and psychiatric disturbances (239).

Eculizumab significantly improved quality of life and provided relief for patients in several complement-driven indications (118). Nevertheless, the monoclonal antibody presents with some unfavorable properties. Besides the increased risk of meningococcal infections due to inhibition of MAC formation, a number of patients remain unresponsive or only achieve an incomplete response, which can be unacceptable in specific indications (147). Furthermore, treatment costs are very high, especially in view of life-long therapy, which has spiked ethical and economical discussions in healthcare (131).

It is evident that many companies are aiming to take a share of the large therapeutic potential of eculizumab in PNH. Second and later generation terminal complement pathway inhibitors are trying to deal with PK challenges, for example through structural modifications, which allow for antibody recycling and a sustained exposure leading to a lower frequency of administration. An improved route of administration and treatment regimen, and eventually a more convenient therapy, would further increase the quality of life of patients (167). Moving from the intravenous to the subcutaneous route opens the possibility of self-administration, which leaves patients less dependent on hospital proximity (211). Moreover, transition from monoclonal antibodies *via* proteins, peptides, and oligonucleotides towards small molecules will gradually ease administration regimens and decreases production efforts (174, 240). A versatile spectrum of mechanisms of action, targets, and target epitopes increases the chance that patients with varying underlying disease causes achieve remission, while the move from general C5 to specific C5a-C5aR1-axis-targeting could be beneficial in avoiding a treatment-induced infection risk.

Besides the above elaborated complement inhibitors in clinical development, several novel agents have been described in preclinical studies that are summarized elsewhere (123). However, usually only sparse data is made available about innovations in the field before clinical development is reached (241). Consequently, the pipeline of terminal complement pathway inhibitors will continue to present novel therapeutic agents in this exciting field providing a plethora of potentially efficacious treatment options to improve patients’ lives in a growing number of rare diseases.

## Author Contributions

All authors listed have made a substantial, direct, and intellectual contribution to the work and approved it for publication.

## Conflict of Interest

MA-O, JD, and PK were employed by the company Idorsia Pharmaceuticals Ltd. The views expressed in this article are those of the authors and do not necessarily reflect those of their company.

The remaining author declares that the research was conducted in the absence of any commercial or financial relationships that could be construed as a potential conflict of interest.
